# [Corrigendum] TIP60 governs the auto-ubiquitination of UHRF1 through USP7 dissociation from the UHRF1/USP7 complex

**DOI:** 10.3892/ijo.2026.5938

**Published:** 2026-09-14

**Authors:** Tanveer Ahmad, Waseem Ashraf, Abdulkhaleg Ibrahim, Liliyana Zaayter, Christian D. Muller, Ali Hamiche, Yves Mély, Christian Bronner, Marc Mousli

Int J Oncol 59: 89, 2021; DOI: 10.3892/ijo.2021.5269

Following the publication of the above article, and an Expression of Concern statement (doi: 10.3892/ijo.2026.5908) that was published in light of the fact that the GAPDH control western blots shown in Fig. 5A on p. 9 were strikingly similar to the GAPDH control western blots shown in Fig. 5D, the authors have responded to the queries raised by the Editorial Office. The GAPDH panels shown in Figs. 5A and 5D were generated from the same experiment and the same set of protein lysates: Fig. 5A presented the analysis of total protein expression, whereas Fig. 5D presented immunoprecipitation analyses performed using material derived from these same experimental samples. Consequently, the GAPDH loading control was legitimately shared between the two figures. However, given that the original figure legend did not explicitly state that the same GAPDH loading control was used for both analyses, to eliminate any possible ambiguity for readers, the authors have revised Fig. 5 by replacing the representative '-MG132 panel' in Fig. 5A with results obtained from an independent repeat experiment performed under identical experimental conditions. The revised version of Fig. 5 is shown on the next page. Note that this revised figure reproduces the original findings, and does not affect the quantitative analyses, the interpretation of the data, or the conclusions of the study.

Regarding a query raised by the Editorial Office concerning the presentation of flow cytometric plots in Fig. 8, the authors explained that Fig. 8E was generated by applying an additional fluorescence gate to the parent flow cytometry dataset shown in Fig. 8B, which represents standard flow cytometric analysis, rather than duplication of experimental data, and so this figure was presented correctly, and as intended by the authors.

The authors thank the Editor of *International Journal of Oncology* for granting them the opportunity to publish this corrigendum, and all the authors agree with its publication.

## Figures and Tables

**Figure 5:**
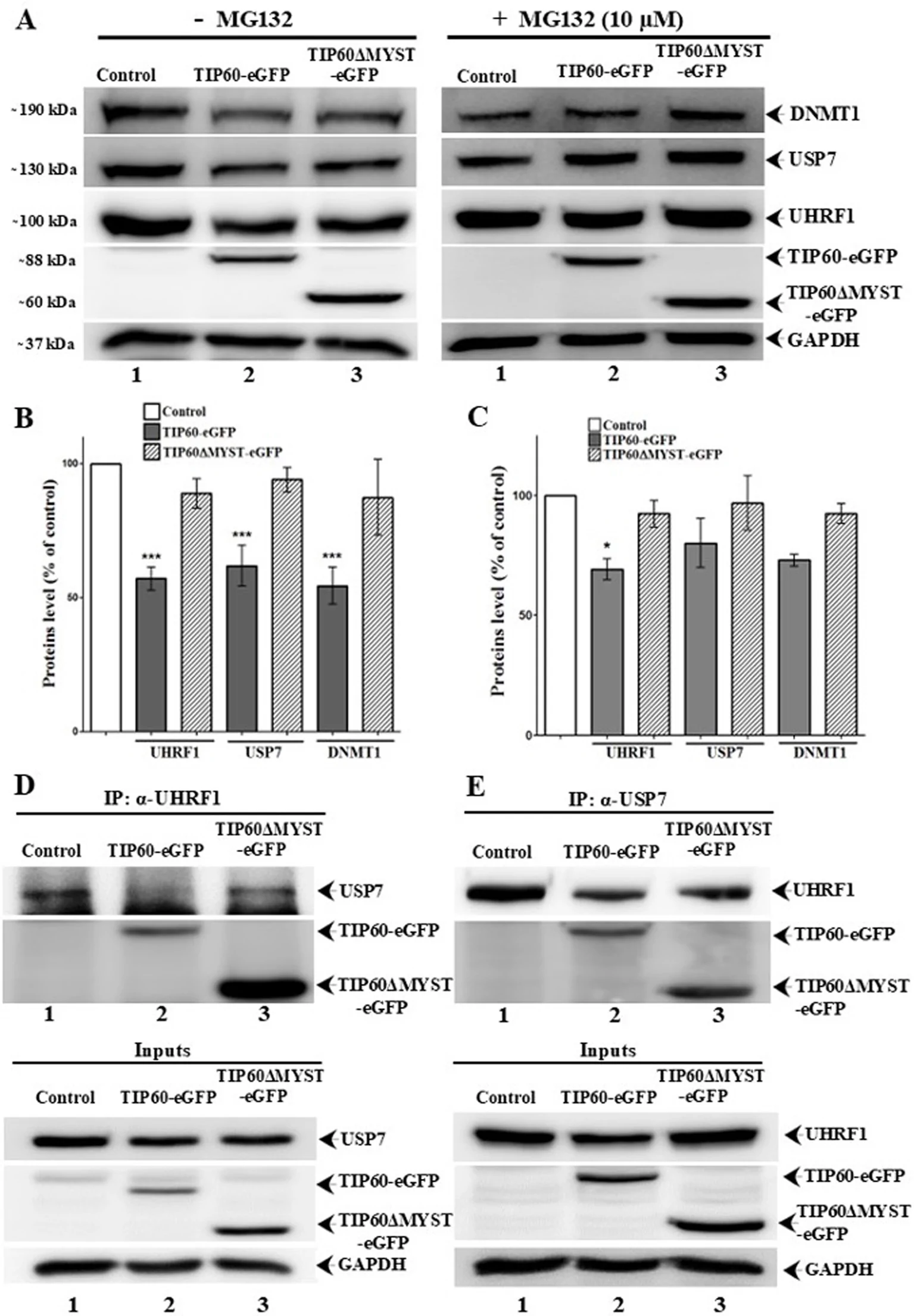
TIP60 interferes with UHRF1-USP7 association and their expression levels. HeLa cells were transfected with either TIP60-eGFP WT or TIP60ΔMYST-eGFP mutant. Western blot and immunoprecipitated samples were resolved by SDS-PAGE and immunoblotted with anti-UHRF1, anti-USP7 and anti-DNMT1 antibodies. (A) Effect of TIP60 on UHRF1, USP7 and DNMT1 levels with or without MG-132 treatment, respectively. (B and C) Quantification of the effect of TIP60 on UHRF1, USP7 and DNMT1 levels with or without MG-132 treatment, respectively. Values are the mean ± SEM from at least three independent experiments which were analyzed statistically by one-way ANOVA with Tukey's post hoc test (^*^P<0.05; ^***^P<0.001 vs. control group). (D) Anti-UHRF1 antibody was used to co-immunoprecipitate UHRF1 and its partner USP7. (E) In a reciprocal experiment, anti-USP7 antibody was used to co-immunoprecipitate USP7 and UHRF1. Inputs and IP gels were processed in parallel under similar conditions. UHRF1, ubiquitin-like, containing PHD and RING finger domains 1; TIP60, Tat interactive protein, 60 kDa; USP7, ubiquitin-specific-processing protease 7; DNMT1, DNA methyltransferase 1.

